# The effect of remimazolam on hypotension risk during procedural sedation

**DOI:** 10.1097/JS9.0000000000001012

**Published:** 2023-12-14

**Authors:** Kuo-Chuan Hung, I-Wen Chen, Ping-Hsin Liu

**Affiliations:** a Department of Anesthesiology, Chi Mei Medical Center; b Department of Anesthesiology, Chi Mei Medical Center, Liouying, Tainan City; c Department of Anesthesiology, E-Da Dachang Hospital, I-Shou University, Kaohsiung City, Taiwan

*Dear Editor,*

We read with interest the recent article by Zhao *et al*.^1^ titled ‘The safety and efficacy between remimazolam and propofol in intravenous anesthesia of endoscopy operation: a systematic review and meta-analysis’ published in the *International Journal of Surgery*. The authors performed a systematic review and meta-analysis of seven randomized controlled trials comparing remimazolam versus propofol for sedation during endoscopic procedures^1^. They found that remimazolam resulted in significantly less hypotension (relative risk 0.45, 95% CI 0.28–0.73, *P*=0.001) and respiratory depression (relative risk 0.20, 95% CI 0.08–0.47, *P*=0.0002) compared to propofol^1^. However, remimazolam was also associated with a slightly lower procedural success rate^1^. Avoiding intraoperative hypotension is important to prevent organ hypoperfusion and ischemia^2^. Although the authors^1^ highlighted the evidence supporting the ability of remimazolam to reduce the risk of hypotension, the finding may still be in its preliminary stages, given the relatively small number of patients (*n*=1109) included in this meta-analysis.

Trial sequential analysis is commonly employed to establish the required sample size and define boundaries for identifying or dismissing the effectiveness of an intervention^3,4^. This method assists in deciding if additional trials are necessary and helps avoid the hasty acceptance of new treatments. To further assess the firmness of evidence, we conducted a trial sequential analysis on the raw data of the previously published meta-analysis^1^ for hypotension outcome. Trial sequential analysis was performed with the TSA viewer version 0.9.5.10 Beta (www.ctu.dk/tsa).

We applied a type 1 error rate of 5%, aimed for a statistical power of 80%, and utilized a relative risk reduction of 20% to establish the superiority of the intervention. As shown in Figure 1, the resultant *z*-curve did not cross the trial sequential monitoring boundary or required information size boundary, indicating that the evidence is still inconclusive. Accordingly, more randomized controlled trials are needed to confirm the hypotension advantage of remimazolam over propofol. In conclusion, while early results seem to favor remimazolam over propofol regarding the risk of hypotension, more definitive randomized controlled trials are warranted before changing clinical practice guidelines.

**Figure 1:**
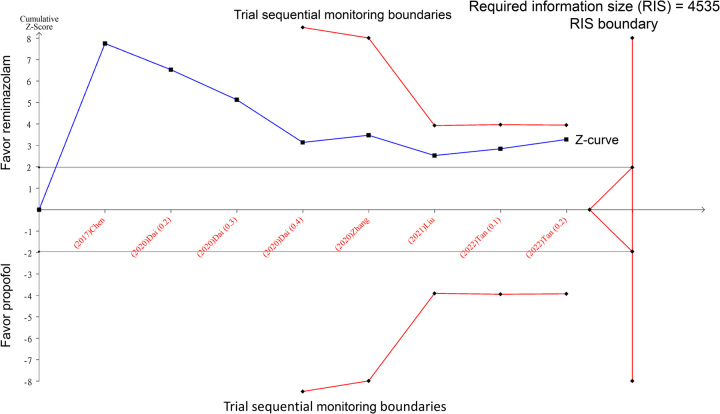
Trial sequential analysis was conducted using an overall type 1 error of 5%, power of 80%, and a hypothetical 20% relative risk reduction. The *z*-curve does not cross the trial sequential monitoring boundary, indicating insufficient evidence accrued till date to confirm lower hypotension risk with remimazolam compared to propofol. Further randomized controlled trials are warranted to precisely estimate the hypotension advantage of remimazolam over propofol for procedural sedation.

## Ethical approval

Not applicable.

## Consent

Not applicable.

## Sources of funding

No external funding was received for this study.

## Author contribution

K.-C.H. and P.-H.L.: wrote the main manuscript text; I-W.C.: prepared Figure 1. All authors read and approved the final version of the manuscript.

## Conflicts of interest disclosure

The authors declare no conflicts of interest.

## Research registration unique identifying number (UIN)

Not applicable.

## Guarantor

Kuo-Chuan Hung.

## Data availability statement

The datasets used and/or analyzed in the current study are available from the corresponding author upon reasonable request.

## Provenance and peer review

This paper was not invited.
